# Twin Circumflex Arteries: A Rare Coronary Artery Anomaly

**Published:** 2018-01

**Authors:** Kahraman Coşansu, Mustafa Tarık Ağaç, Harun Kılıç, Ramazan Akdemir, Hüseyin Gündüz

**Affiliations:** *Department of Cardiology, Sakarya University Training and Research Hospital, Sakarya, Turkey.*

**Keywords:** *Coronary angiography*, *Coronary vessels*, *Coronary vessel anomalies*

## Abstract

A circumflex artery originating from an ostium apart from the left main artery is one of the most common coronary artery anomalies. However, a dual origin of the circumflex artery is an extremely rare anomaly. We describe a 55-year-old male patient admitted to our clinic with the diagnosis of unstable angina. Angiography revealed twin circumflex arteries: one from the left main artery and the other from the proximal right coronary artery and a stenotic left anterior descending coronary artery (LAD). The patient was treated with percutaneous coronary intervention on the LAD lesion. His overall condition was good at 2 weeks’ follow-up.

## Introduction

Coronary artery anomalies are rare in the general population, with an incidence rate of about 1%, and most of them are found incidentally during coronary angiography.^1^^, ^^2^ One of the most common coronary anomalies is a circumflex (Cx) coronary artery anomalously originating from the right sinus of Valsalva; nevertheless, double Cx arteries originating from the left and right coronary systems constitute a type of anomaly rarely reported in the literature.^3^^, ^^4^ We present a case of twin Cx arteries: one from the left main artery and the other from the proximal right coronary artery.

## Case Report

**Figure 1:**
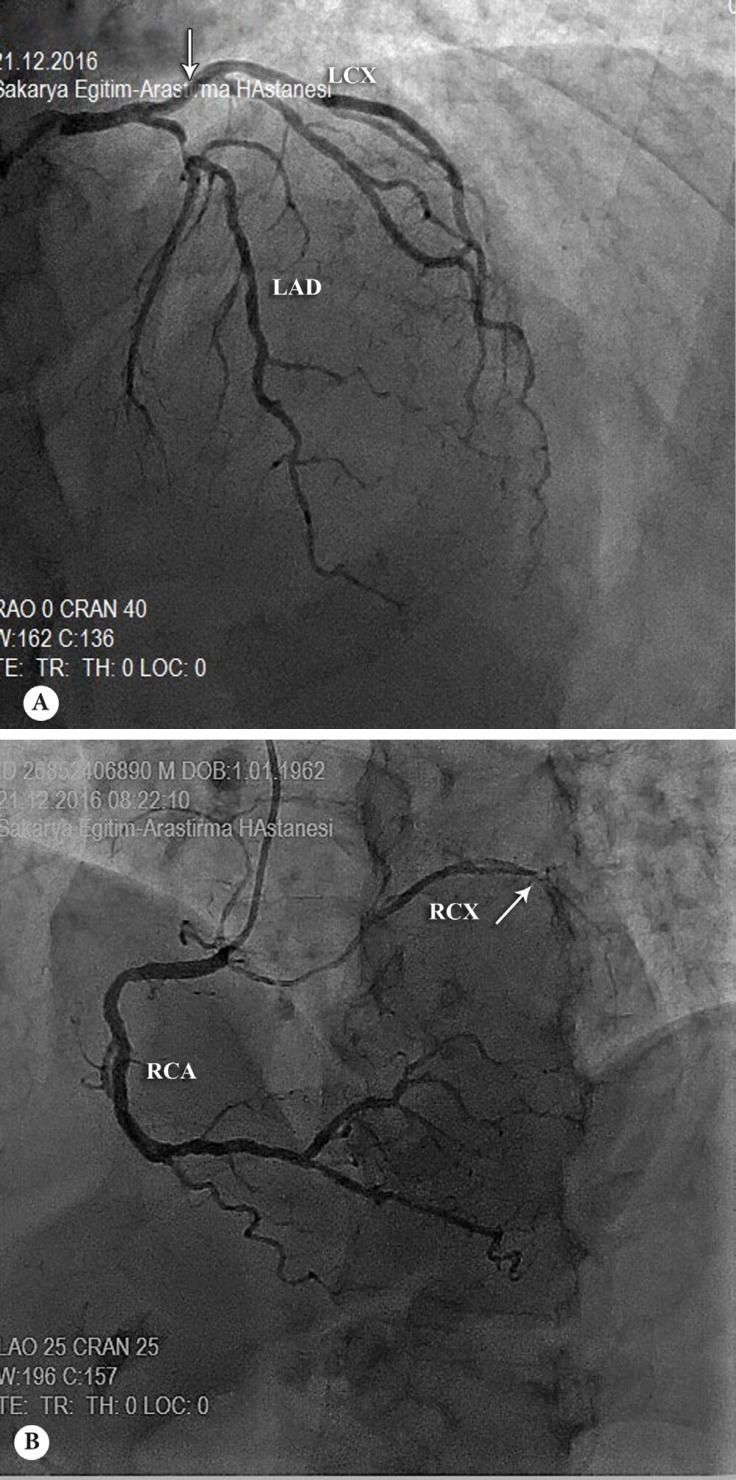
Coronary angiogram in the right anterior oblique cranial view (A), showing the left circumflex artery (LCx) (arrow) and the left anterior descending artery (LAD) originating from the left main coronary artery. When the right coronary ostium was cannulated (B), another circumflex artery (RCx) (arrow) was noticed in addition to a right coronary artery (RCA) in the left anterior oblique cranial view.

A 55-year-old man with a history of chest discomfort was referred to our clinic. The patient reported that he had angina of 6 months’ duration, but his angina had changed from the Canadian Cardiovascular Society (CCS) I-II to CCS III over the preceding 2 days. His physical examination, echocardiogram, and electrocardiogram reports were all normal. Following physical examination and initial tests, a diagnostic coronary arteriography was performed. Conventional angiography revealed that the left anterior descending coronary artery (LAD) had a critical proximal lesion and the left Cx (LCx) was normal and originated from the left main coronary artery (Figure 1). Additionally, there was another nondominant Cx (RCx) arising from the proximal part of the right coronary artery with a significant diffuse stenosis (Figure 1). There was also 35% stenosis in the distal left main coronary artery. An EBU guiding catheter was used to cannulate the left main ostium and the target lesion was passed using a 0.014″ guide wire. Thereafter, stenting was successfully performed with a 2.25 × 16 mm drug-eluting stent for the LAD lesion. The patient’s symptoms were relieved after the successful intervention on the LAD. He was discharged on the postoperative day in good condition. He came to our clinic for control after 2 weeks and reported that he had not experienced any angina since his discharge.

## Discussion

Normally, the left main coronary artery originates from the left sinus of Valsalva and gives rise to the LAD and Cx arteries. The Cx artery courses in the left atrioventricular groove and provides the first obtuse marginal branch.^5^ The most frequently found anomalies include a Cx artery with a separate origin of the LAD and Cx arteries, followed by a Cx artery arising from the right sinus of Valsalva or the right coronary artery.^6^ There are only a few cases of twin Cx arteries originating from both left and right coronary systems that have been reported in the literature. The anomalous origin of the Cx artery from the proximal right coronary artery or from the right sinus of Valsalva was first described by Antopol and Kugal^7^ in 1933. There have been only 7 previously reported cases of bilaterally arising twin LCx arteries: one LCx artery arose from the left main coronary artery and the others from the right aortic sinus (3 cases) or the ostial of the right coronary artery (4 cases) following thereafter a retroaortic course to the left.^8^^-^^14^ In 2008, Attar et al.^8^ reported a case of twin Cx arteries: one from the left main artery and the other from the right coronary sinus. Along the same lines, Van der Velden et al.^4^ presented a case with the coexistence of coronary fistulae and twin Cx arteries. Elsewhere, Karabay et al.^9^ presented a case of twin Cx arteries arising from the left and right coronary systems with acute inferior myocardial infarction treated via percutaneous coronary intervention. In a study by Cicek et al.,^10^ there were significant stenoses at both of the twin Cx arteries, leading to heart failure. Andreou et al.^12^ reported a case for preoperative identification of this anomaly in patients undergoing aortic valve surgery. Another investigation by Otlu et al.^14^ reported transradial percutaneous coronary intervention in a patient with twin Cx arteries. In this case report, we described a patient with twin Cx arteries: one (LCx) originating from the left main coronary artery and the other one, a nondominant Cx (RCx), arising from the proximal part of the right coronary artery.

Some coronary artery anomalies may cause chest pain, heart failure, arrhythmia, and sudden death. These manifestations may be in consequence of the repeated compression of the anomalous artery by a dilated aortic root or of slit-like ostia or of unusual angling as a result of the retroaortic course of the Cx.^15^ Myocardial ischemia can occur because of earlier and more aggressive atherosclerosis compared to a normal coronary artery,^16^ found exclusively in anomalous vessels arising from the right side.^17^ Click et al.^18^ confirmed that the incidence of stenosis was greater in the Cx arteries originating from the right coronary sinus than that in normal Cx arteries originating from the left main coronary artery. Nonetheless, this coronary anomaly has generally been classified as benign and may be asymptomatic in the majority of patients.^19^ In our patient, an anomalous nondominant Cx artery had significant diffuse stenosis, while the original Cx was normal. The patient’s symptoms were relieved after successful intervention on the LAD; we, therefore, did not consider any intervention on the anomalous Cx.

To our knowledge, an aberrant accessory but normal Cx artery has no clinical significance. Nevertheless, the clinical significance of the anomaly may be important in patients undergoing coronary intervention or cardiac surgery.^20^ It is important to inform the surgeons so as to avoid accidentally cross-clamping or transecting the artery during surgery. An abnormal course of the artery (retroaortic) is also a cause for concern.

## Conclusion

The most important problem in diagnosing double Cx arteries is the separate origin of the 2 Cx arteries from different ostia on the left or right aortic sinus of Valsalva. Thus, the angiographer must always keep in mind this possibility.

## Notes:

**This paper should be cited as:** Coşansu K, Ağaç MT, Kılıç H, Akdemir R, Gündüz H. Twin Circumflex Arteries: A Rare Coronary Artery Anomaly. J Teh Univ Heart Ctr 2018;13(1):32-34.
